# The Role of MDR1 (C3435T) Gene Polymorphism in Patients with Chronic Obstructive Pulmonary Disease Associated with Type 2 Diabetes Mellitus

**DOI:** 10.25122/jml-2020-0139

**Published:** 2020

**Authors:** Natalia Vasylivna Chernetska, Hanna Yaroslavivna Stupnytska, Oleksandr Ivanovich Fediv

**Affiliations:** 1. Department of Internal Medicine and Infectious Diseases, Higher State Educational Establishment of Ukraine “Bukovinian State Medical University”, Chernivtsi, Ukraine; 2. Department of Propaedeutic of Internal diseases, Higher State Educational Establishment of Ukraine “Bukovinian State Medical University”, Chernivtsi, Ukraine

**Keywords:** Chronic obstructive pulmonary disease, diabetes mellitus, MDR1 gene

## Abstract

Chronic obstructive pulmonary disease is a multifactorial disease characterized by gene-gene interaction as well as environmental effects. The incidence of type 2 diabetes mellitus is proved to be higher in the presence of chronic obstructive pulmonary disease than in the case of its absence.

We aimed to study the genotypes of MDR1 (C3435T) gene polymorphism and its relationship with clinical, instrumental, and laboratory parameters in chronic obstructive pulmonary disease associated with type 2 diabetes mellitus.

All the patients were divided into two groups. The first group included 53 patients with chronic obstructive pulmonary disease, and the second group included 49 patients with chronic obstructive pulmonary disease with comorbid type 2 diabetes mellitus. The COPD assessment test (CAT), 6-minute walk test, BODE integral index, spirometry, and bioimpedansometry were used for examination. Lipid spectrum, carbohydrate metabolism, endothelial functional status, leptin, adiponectin, and serum levels were also determined by means of enzyme immunoassay.

Our study results showed no significant difference between the genotypes of the control group of healthy individuals and patients with chronic obstructive pulmonary disease and comorbid type 2 diabetes mellitus. Though, a certain association of this gene polymorphism with clinical findings by CAT-test, specific parameters of carbohydrate (fasting glucose) and lipid metabolism (total cholesterol and low-density cholesterol lipoproteins), endothelial functional state (nitrate/nitrite level) with the minor allele T available was found.

## Introduction

Chronic obstructive pulmonary disease (COPD) is known to be a multifactorial disease [1-5] characterized by gene-gene interaction [6-8], as well as environmental impact [9, 10]. However, not all the mechanisms for the development and advance of this pathology have been fully understood. The development of chronic systemic inflammation is found to be the characteristic of COPD and the appearance of systemic effects [11], leading to an increased incidence of concomitant pathology [12, 13]. The incidence of type 2 diabetes mellitus (DM) with COPD is proved to be higher than in the case of its absence [14-16]. The role of the MDR1 gene (C3435T) polymorphism in the development and advance of COPD [17-19] has been studied, although the results of these studies are controversial. The multiple drug resistance gene (MDR1) is known to be localized on chromosome 7q21, and the products of this gene (multiple drug resistance protein-1 and resistance protein associated with lung) act as antioxidants, protecting the lung tissue from oxidative stress, toxins, and poisons released when smoking cigarettes [11].

This gene polymorphism has also attracted the attention of scientists in the study of type 2 diabetes mellitus development mechanisms [20]. Although the results obtained are single and ambiguous, they can be used to correct lipid and carbohydrate metabolism disorders, taking into account pharmacogenetic aspects.

Therefore, the study of MDR1 gene polymorphism associated with COPD and type 2 diabetes mellitus appears reasonable.

We aimed to study the genotypes of MDR1 (C3435T) gene polymorphism and its relationship with clinical, instrumental, and laboratory parameters in the case of chronic obstructive pulmonary disease associated with type 2 diabetes mellitus.

## Material and Methods

Fifty-three patients with COPD and 49 patients with COPD with concomitant type 2 diabetes were examined. All patients were in remission of the disease and met the inclusion and exclusion criteria. COPD and type 2 diabetes mellitus were diagnosed according to international protocols. The study meets the requirements of the Helsinki Declaration of the World Medical Association “Ethical principles for medical research involving human subjects as the object of study” and the Committee on Bioethics of Higher State Educational Establishment of Ukraine “Bukovinian State Medical University” approved this study (No. 2/2016). The diagnosis and stage of COPD were established following the GOLD 2017 recommendations, by order of the Ministry of Health of Ukraine No. 555 of 27.06.2013 and changes to the Order No. 270 of April 16, 2014. The clinical characteristics of the patients are illustrated in Table 1.

**Table 1:** Clinical characteristics of patients.

**Parameters**	**COPD (1^st^ group)**	**COPD and type 2 diabetes mellitus (2^nd^ group)**	**p**
**Gender, male/female**	48/5	40/9	p>0.05
**Age**	64.55 ± 1.51	60.67 ± 1.22	p>0.05
**Index of pack-years**	32.05 ± 3.41	17.39 ± 2.82	p>0.001
**Duration of COPD**	11.92 ± 0.89	9.16 ± 0.71	p>0.05
**BMI**	23.94 ± 0.54	34.09 ± 0.61	p>0.001
**FEV1, % of proper value**	46.42 ± 1.89	40.30 ± 1.86	p>0.05
**CAT, points**	18.14 ± 1.04	19.55 ± 1.23	p>0.05
**BODE index points**	4.02 ± 0.28	5.36 ± 0.30	p>0.05

The COPD assessment test (CAT), 6-minute walk test, BODE integral index, and spirometry (“BTL08 SpiroPro” Spirograph, UK) were used in the study, which included patients with COPD with an FEV1/FVC ratio less than 0.7 and with II-m, III and IV degrees of bronchial obstruction according to the GOLD spirometric classification.

Bioimpedance analysis (BC-601 Portable Apparatus, TANITA, Japan) was used to assess body composition. The body weight, body mass index (BMI), muscle mass, fat percentage, visceral fat level, and water percentage in the body were determined.

Blood lipid spectrum was examined for total cholesterol (TC), triglycerol (TG), low-density lipoprotein (LDL) cholesterol, very-low-density lipoprotein (VLDL) and high-density lipoprotein (HDL) cholesterol levels (PZCormay, Poland). Carbohydrate metabolism was studied using the fasting blood glucose test, glycosylated hemoglobin (HbA1c), and insulin levels.

The level of glycemia was investigated by the glucose oxidase method using standard sets of reagents manufactured by NPP “Philitis Diagnostics” (Ukraine). Glycosylated hemoglobin was determined using a photocolorimetric method using a set of reagents manufactured by ErbaLachemas. r. o (Czech Republic). The level of immunoreactive insulin (IRI) was examined by enzyme-linked immunosorbent assay using DRG International, Inc reagents (USA). The functional state of the endothelium was examined for the content of stable metabolites of nitrogen monoxide (nitrites/nitrates), endothelin-1 (ET-1) in the blood, the number of fused circulating endothelial cells (CEC), the content of soluble adhesion molecule (sVCAM-1) [1]. The number of circulating endothelial cells in the blood was determined by the J. Hladovec method (1978) in the modification of N. Petrischev and others (1999). Blood content of stable metabolites of NO (nitrites/nitrates) was investigated using the L. C. Greenetal method (1982), ET-1 level - by enzyme-linked immunosorbent assay using “BiomedicaMedizinprodukteGmb Hand Co KG” reagents (Austria). sVCAM-1 levels were determined in serum by ELISA using BenderMedSystems reagents (Austria). Serum levels of leptin (Diagnostics BiochemCanadaInc, Canada), adiponectin (Assay, USA), and resistin (Mediagnost, Germany), TNFα and TGFβ1 (Bender Med Systems GmbH, Austria) were determined using enzyme immunoassay kits. Serum CRP levels were determined according to the instructions (Humatex CRP “HUMAN”, Germany).

Genomic DNA was isolated from the peripheral blood for molecular genetic studies. Genotyping of the polymorphic variant C3435T of the MDR1 gene was done according to the protocol of Turgut S. et al. Two-sided Pearsonah-square test (χ2) was used to estimate the distribution of genotypes and alleles between groups. The calculations were performed using the Statistica software, version 10.0 (Stat Softinc, USA).

## Results

The frequency of the genotype of the MDR1 gene (C3435T) polymorphism in control and COPD patients without and with comorbid type 2 diabetes mellitus is presented in Table 2. There were no differences between the genotypes in the presented groups.

**Table 2:** Genotypes of the MDR1 (С3435Т) gene polymorphism.

C3435T	Control group (practically visual)n=40	Patients with COPD n=53	Patients with COPD and type 2 diabetes mellitus n=49
	Genotypes		
**CC**	9 (22.50%)	15 (28.3%)	13 (26.53)
**CT**	19 (47.50 %)	26 (49.05%)	23 (46.94%)
**TT**	12 (30%)	12 (22.64%)	13 (26.53%)

The analysis of bioimpedansometry indicators (Table 3) demonstrated a significant difference between the group of COPD patients and COPD with concomitant type 2 diabetes mellitus in BMI, fat percentage, visceral fat, bone mass regardless of the MDR1 gene polymorphism type 35 (C34). Muscle mass in these groups of patients was significantly different for genotypes CC and CT than the genotype TT, which was not reliable (p> 0.05). Forced expiratory volume per second (FEV1) in both groups of patients had no significant difference and did not depend on the genotype of MDR1 gene polymorphism (C3435T).

**Table 3:** Parameters of bioimpedansometry and the function of external respiration in patients with COPD with concomitant type 2 diabetes mellitus depending on the MDR1 (С3435Т) gene polymorphism.

Parameters	Genotype	Patients with COPD and type 2 diabetes mellitus	Patients with COPD	P_A-B_
**BMI, kg/m^2^**	CC CT TT	35.08 ± 1.30 34.10 ± 0.82 33.09 ± 1.28	23.48 ± 1.04 23.93 ± 0.78 24.53 ± 1.10	p<0.001 p<0.001 p<0.001
**% fat content in the body**	CC CT TT	29.16 ± 1.34 32.32 ± 7.76 30.28 ± 2.51	19.34 ± 2.57 20.21 ± 1.93 20.60 ± 3.12	p=0.01 p<0.001 p=0.01
**Muscle mass, kg**	CC CT TT	66.68 ± 2.23 61.92 ± 2.69 61.39 ± 2.46	50.93 ± 2.08 52.55 ± 2.86 54.55 ± 2.86	p<0.001 p<0.05 p>0.05
**The level of visceral fat**	CC CT TT	17.31 ± 1.30 15.44 ± 0.71 15.23 ± 0.85	10.20 ± 0.77 10.50 ± 0.62 10.33 ± 0.74	p<0.001 p<0.001 p<0.001
**Bone mass**	CC CT TT	3.52 ± 0.12 3.21 ± 0.13 3.21 ± 0.12	2.75 ± 0.11 2.80 ± 0.10 2.80 ± 0.10	p<0.001 p<0.001 p<0.05
**% water in the body**	CC CT TT	51.05 ± 1.88 49.09 ± 1.80 52.34 ± 2.10	58.08 ± 2.29 57.47 ± 1.71 57.98 ± 2.59	p<0.05 p<0.05 p>0.05
**VFE_1_, % from the proper value**	CC CT TT	37.25 ± 3.99 40.64 ± 2.30 43.37 ± 4.35	45.68 ± 3.77 48.14 ± 12.72 43.97 ± 4.29	p>0.05 p>0.05 p>0.05

When performing the CAT test (Table 4), it was found that in the group of COPD patients with concomitant type 2 diabetes mellitus, the total score of the TT genotype exceeded that of the CC genotype by 31.4% (p <0.05). The 6-minute walk test showed the difference between the COPD patient group and its comorbid course with type 2 diabetes mellitus in the CT genotype (patients walked 23.1% less in the first group). The Bode integral index was significantly higher in COPD patients with concomitant type 2 diabetes mellitus than that of the CT genotype (28.7%, p <0.05).

**Table 4:** CAT indices, shortness of breath, 6-minute distance walk test in COPD patients with concomitant type 2 diabetes mellitus, depending on the MDR1 (С3435Т) gene polymorphism.

Indices	Genotype	Patients with COPD and type 2 diabetes mellitus	Patients with COPD	P_A-B_
**CAT, points**	CC CT TT	16.15 ± 2.31 19.70 ± 1.58 23.54 ± 2.79 **p_CC_<0.05**	14.73 ± 1.37 18.04 ± 1.54 18.33 ± 1.75	p>0.05 p>0.05 p>0.05
**Scale of shortness of breath, points**	CC CT TT	2.54 ± 0.27 1.91 ± 0.27 2.08 ± 0.33	2.33 ± 0.33 2.12 ± 0.24 2.58 ± 0.38	p>0.05 p>0.05 p>0.05
**6-minute walk test**	CC CT TT	298.846 ± 24.496 275.652 ± 15.965 300.000 ± 34.530	292.000 ± 20.387 339.423 ± 18.446 327.083 ± 27.978	p>0.05 p>0.05 p>0.05
**BODE index**	CC CT TT	5.73 ± 0.45 5.19 ± 0.44 5.25 ± 0.73	4.38 ± 0.63 3.70 ± 0.41 4.23 ± 0.50	p>0.05 p<0.05 p>0.05

The interpretation of the results of the carbohydrate metabolism study (Table 5) illustrated that in patients with COPD and comorbid type 2 diabetes mellitus, fasting glucose was significantly higher than that for TT genotype compared to the CC genotype (18.9%, p <0.05).

**Table 5:** Carbohydrate metabolism indices in patients with COPD with concomitant type 2 diabetes mellitus depending on the MDR1 (С3435Т) gene polymorphism.

Indices	Genotype	Patients with COPD and 2^nd^ type 2 diabetes (1^st^ group)	Patients with COPD (2nd group)	P^A-B^
**Fasting glucose, mmol/l**	CC CT TT	6.71 ± 0.68 7.44 ± 0.45 8.27 ± 0.79 **p_CC_<0.05**	4.58 ± 0.22 4.44 ± 0.15 4.66 ± 0.21	p>0.05 p>0.001 p>0.001
**Glucose after 2 years, mmol/l**	CC CT TT	8.97 ± 1.31 8.59 ± 0.93 9.77 ± 1.34	6.15 ± 0.86 5.44 ± 0.25 5.34 ± 0.18	p>0.05 p>0.05 p>0.05
**HbA1C, %**	CC CT TT	6.87 ± 0.63 6.01 ± 0.34 6.02 ± 0.45	5.09 ± 0.36 4.71 ± 0.13 4.79 ± 0.25	p>0.05 p>0.05 p>0.05
**Insulin on an empty stomach, μUn / l**	CC CT TT	20.73 ± 3.93 20.24 ± 5.17 25.43 ± 5.96	4.90 ± 0.34 2.78 ± 0.28 2.64 ± 0.42	p>0.05 p<0.05 p>0.05

The level of total cholesterol (Table 6) in patients with COPD with concomitant type 2 diabetes mellitus was found to be significantly higher for the TT genotype (12.9%) compared with the group with the CC genotype (p <0.05). LDL cholesterol was also significantly higher in patients of the first group with the genotype TT than the CC and CT genotype (by 13.75 and 12.88%, respectively, p <0.05).

**Table 6:** Lipid metabolism indices in patients with COPD and concomitant type 2 diabetes mellitus depending on the MDR1 (C3435T) gene polymorphism.

**Indices**	**Genotype**	**COPD patients with type 2 diabetes mellitus**	**COPD patients**	**P_A-B_**
**Total cholesterol, mmol/l**	CC CT TT	6.43 ± 0.25 6.84 ± 0.30 7.38 ± 0.34 p_CC_<0.05	6.03 ± 0.33 5.55 ± 0.20 5.56 ± 0.33	p>0.05 p>0.05 p>0.05
**Triglycerols, mmol/l**	CC CT TT	2.78 ± 0.22 2.39 ± 0.15 2.76 ± 0.18	1.83 ± 0.12 1.88 ± 0.11 1.89 ± 0.23	p>0.05 p>0.05 p>0.05
**Low density cholesterol lipoprotein, mmol/l**	CC CT TT	69.00 ± 3.50 69.7 ± 3.40 80.00 ± 3.51 p_CC_<0.05 p_CT_<0.05	62.53 ± 3.62 58.27 ± 1.71 57.65 ± 2.99	p>0.05 p>0.05 p>0.001
**High density cholesterol lipoprotein, mmol/l**	CC CT TT	0.97 ± 0.04 1.03 ± 0.06 0.84 ± 0.04	1.17 ± 0.10 1.31 ± 0.09 1.19 ± 0.11	p>0.05 p<0.05 p>0.05
**Very low-density cholesterol lipoprotein, mmol/l**	CC CT TT	1.25 ± 0.10 1.08 ± 0.07 1.24 ± 0.08	0.82 ± 0.06 0.84 ± 0.05 0.85 ± 0.10	p<0.001 p<0.05 p<0.05

Analysis of systemic inflammation (Table 7) found that TNFα level was likely to be higher in COPD patients with concomitant type 2 diabetes mellitus, regardless of genotype. Although the C-reactive protein (CRP) level was higher in COPD patients with concomitant type 2 diabetes mellitus compared with the second group, the difference was significant only in the CC genotype (p <0.05). TGF was not significantly different between the two groups of patients.

**Table 7:** Indices of cytokine profile and C-reactive protein in patients with COPD with concomitant type 2 diabetes mellitus depending on the MDR1 (C3435T) gene polymorphism.

Indices	Genotype	COPD patients with type 2 diabetes mellitus	COPD patients	**P_A-B_**
**Leptin, ng/ml**	CC CT TT	55.35 ± 9.26 56.32 ± 5.95 43.55 ± 10.88	20.56 ± 3.80 17.10 ± 5.36 12.14 ± 1.71	p>0.05 p>0.001 p>0.05
**Resistin, ng/ml**	CC CT TT	14.06 ± 1.66 14.22 ± 1.19 11.82 ± 5.27	8.61 ± 0.80 8.76 ± 2.52 8.19 ± 0.79	p=0.001 p<0.05 p>0.05
**Adiponectin, ng/ml**	CC CT TT	5.57 ± 0.46 4.75 ± 0.43 3.89 ± 0.35	7.98 ± 1.38 10.39 ± 1.25 9.00 ± 2.30	p>0.05 p<0.001 p=0.001
**TNF_a_, pg/ml**	CC CT TT	727.73 ± 38.54 673.71 ± 53.01 545.47 ± 75.89	346.51 ± 83.73 491.95 ± 71.72 214.90 ± 52.18	p<0.001 p<0.05 p<0.05
**TGF_b_1, pg/ml**	CC CT TT	15980.28 ± 2143.11 14952.76 ± 1167.00 16599.69 ± 1584.24	11449.91 ± 1840.91 13290.08 ± 2516.61 15669.53 ± 1516.55	p>0.05 p>0.05 p>0.05
**C-reactive protein**	CC CT TT	5.95 ± 0.84 7.73 ± 0.76 8.72 ± 1.03	4.40 ± 0.74 6.87 ± 0.77 **p_CC_<0.05** 6.42 ± 1.29	p<0.05 p>0.05 p>0.05

Serum leptin levels were significantly higher in COPD patients with concomitant type 2 diabetes mellitus, regardless of genotype. The level of resistin was probably higher in the latter group of patients than those with the CC and CT genotypes, but the TT genotype had no confidence between the two groups. Adiponectin was significantly lower in COPD patients with concomitant type 2 diabetes mellitus in the presence of the T allele.

When examining endothelial functional status indicators (Table 8), it was found that CEC was significantly higher in patients with COPD and comorbid type 2 diabetes mellitus regardless of genotype. However, the level of nitrates/nitrites in the first group of patients was 41.5% lower for the TT genotype compared to the CC genotype (p <0.05). The level of ET-1 in patients with COPD with concomitant type 2 diabetes mellitus was significantly higher regardless of genotype. VCAM-1 levels were significantly higher in this group compared to the CC and CT genotype (p <0.05).

**Table 8:** Indices of endothelial functional status in COPD patients with concomitant type 2 diabetes mellitus depending on the MDR1 (C3435T) gene polymorphism.

Indices	Genotype	COPD patients with type 2 diabetes mellitus	COPD patients	P_A-B_
**Number of circulating endothelial cells, 10^4^/l**	CC CT TT	19.23 ± 1.10 19.71 ± 1.18 18.33 ± 1.38	11.27 ± 1.08 11.88 ± 0.89 11.33 ± 1.50	p<0.05 p<0.05 p<0.05
**Nitrate/nitrite level, μmol/l**	CC CT TT	17.13 ± 1.17 14.17 ± 0.96 12.11 ± 1.17 **p_CC_<0.05**	17.26 ± 1.84 18.62 ± 1.43 19.49 ± 2.37	p>0.05 p<0.05 p<0.05
**Endothelin-1, pmol/l**	CC CT TT	0.28 ± 0.04 0.27 ± 0.02 0.29 ± 0.03	0.17 ± 0.03 0.16 ± 0.07 0.16 ± 0.07	p<0.05 p<0.05 p<0.05
**VCAM-1, ng/ml**	CC CT TT	3313.18 ± 238.95 3249.34 ± 246.42 3300.00 ± 313.10	2181.38 ± 673.38 1866.20 ± 251.89 2053.00 ± 552.33	p<0.05 p=0.001 p>0.05

## Discussion

Certain scientists have evidenced the role of MDR1 (C3435T) polymorphism in the development and progression of COPD [18]. Thus, Turkish scientists have shown an association between the development of right ventricular dysfunction and oxidative stress with the T allele in COPD patients [17, 19]. The role of this gene polymorphism in the development of type 2 diabetes mellitus is also studied [20]. Thus, Yücel et al. were the first to study the relationship between MDR1 (C3435T) polymorphism and type 2 diabetes mellitus, as well as its effect on blood lipid levels. They showed that for this gene polymorphism, no association of the T allele with disease development and lipid dependence on genotype was detected. Although they did not find any strong direct correlation between the level of lipids in patients with diabetes and MDR1 gene polymorphism (C3435T), they indicate that a connection is possible due to the complex effects of this polymorphism and the use of drugs that patients regularly take [19]. Rizvi et al. also studied the relationship of this gene polymorphism with carbohydrate and lipid metabolism in diabetic patients, and their findings confirmed the previous results [20]. According to the results of their study, there was no difference in genotypes between the groups of patients and the group of healthy individuals in the comorbid course of COPD and type 2 diabetes mellitus. However, in case of association of MDR1 gene (C3435T) polymorphism in COPD patients with concomitant type 2 diabetes mellitus and clinical findings, in particular, according to the results of the CAT test in the TT genotype, the patients obtained a higher number of points. Patients’ exercise tolerance for the 6-minute walk test was also lower in patients with comorbid pathology and showed a significant difference in the CT genotype. The BODE integral index, which is now used to assess not only the prognosis but also the severity of COPD and treatment efficacy, was also higher in COPD patients with type 2 diabetes mellitus than those with the CT genotype. Carbohydrate and lipid metabolism in the comorbid course of disease differed between genotypes. Fasting glucose and total cholesterol levels were significantly higher in the TT genotype compared to the CC genotype. Also, LDL cholesterol was higher in the TT genotype compared to the CC and CT genotype. The indicators of systemic inflammation and adipocytokines in the group of patients with comorbid pathology did not have a significant difference between the genotypes. However, compared with the group of patients with COPD, there was a significant difference between the genotypes, especially for the CC genotype.

Considering that endothelial dysfunction plays one of the leading roles in the pathogenesis of COPD and type 2 diabetes mellitus, association of MDR1 gene (C3435T) polymorphism with indicators of endothelial functional status has been studied. They have been found to be significantly worse in the group of patients with comorbid pathology compared to the group of COPD patients. Simultaneously, the level of nitrates/nitrites in patients with COPD with concomitant type 2 diabetes mellitus was significantly lower in the TT genotype compared to the CC genotype.

## Conclusions

Therefore, the results of our study are indicative of the fact that there is no significant difference between the genotypes of the control group of healthy individuals and patients with COPD and comorbid type 2 diabetes mellitus. However, a certain association of this gene polymorphism with clinical findings has been established on the base of the CAT test, specific indices of carbohydrate (fasting glucose), and lipid metabolism (total cholesterol and LDL cholesterol), endothelial functional state (nitrate/nitrite level) with the T allele. Further investigation is required, and possible use of the findings obtained could be implemented in a personalized treatment in case of comorbidity of chronic obstructive pulmonary disease and type 2 diabetes mellitus, taking into account the MDR1 (S3435T) gene polymorphism.

## Acknowledgment

The study is a fragment of the planned research work of the Department of Internal Medicine and Infectious Diseases of the Higher State Educational Establishment of Ukraine “Bukovinian State Medical University” – “Molecular-genetic and clinical-pathogenetic features of the combined pathology of the internal organs, the role of infectious, metabolic factors in its development, differentiated approaches to treatment” (state registration number 0117U00235).

## Conflict of Interest

The authors declare that there is no conflict of interest.
